# Physical Characteristics of Amorphous and Crystalline Coconut Sugar Powder with the Addition of Tricalcium Phosphate (TCP) as an Anticaking Agent

**DOI:** 10.1155/2020/5320173

**Published:** 2020-09-14

**Authors:** Bambang Nurhadi, Nandi Sukri, Rudy Adi Saputra, Fatonah Isnaini Wandhani, Afifah Indah Nurlita

**Affiliations:** Faculty of Agric-Industrial Technology, Universitas Padjadjaran, Sumedang, Indonesia

## Abstract

The coconut sugar powder produced by vacuum drying and conventional method has high hygroscopicity due to its high sugar content (mostly sucrose). Therefore, it is easier for caking to occur during storage. An anticaking agent such as tricalcium phosphate was therefore added to the powder to maintain its stability. The purpose of this research was to determine the physical characteristics of amorphous and crystalline coconut sugar after the addition of tricalcium phosphate (TCP) in different concentrations. The two types of coconut sugar were prepared by the conventional method, which gave it a predominantly crystalline structure, and the vacuum drying method, which gave it a mainly amorphous structure. The TCP at concentrations 0, 0.5%, and 1% was added to both types of the coconut sugar. The addition of the anticaking agent affected the water sorption of coconut sugar by decreasing the monolayer water content for both types of coconut sugar. TCP seemed to give more significant effect on decreasing the hygroscopicity of crystalline coconut sugar than the amorphous one, while similar trends were obtained in increasing flow ability of both types of coconut sugar. The capacity of TCP to cover the surface of the host coconut powder was proposed as the mechanism of TCP in decreasing hygroscopicity and increasing flow ability of the host powder.

## 1. Introduction

Coconut sugar is commonly produced from the evaporation of coconut sap (called as neera). Neera is the sweet, oyster white-coloured sap liquid tapped from the immature inflorescence of coconut. Neera is obtained from the immature inflorescence of a coconut which is about to burst, and the tapping could be done for 12 to 15 times [1]. The main composition of neera is sucrose with the amount more than 80% (per total solid) and followed by a tiny amount of glucose and fructose (about 2.3% per total solid) [2]. Coconut sugar powder is produced conventionally by heating the coconut sap until reaching a saturated solution, and crystalline coconut sugar powder finally is formed. Coconut sugar was also produced by drying the coconut sap using spray drying and vacuum drying [2]. The dried coconut sugar produced had a mainly amorphous structure in contrast with the crystalline structure of coconut sugar obtained with the conventional method [2].

In drying of coconut sugar, maltodextrin as drying aid material was added to increase its anhydrous glass transition temperature higher than ambient temperature. The addition of maltodextrin which has a high glass transition temperature might increase process stability and storage of solid food in reducing caking phenomena and stickiness and increasing the flow ability. The addition of maltodextrin in the ratio of 50% (from total solid) was needed to create a significant impact on glass transition temperature in producing coconut sugar powder with vacuum drying [2]. Both the types of coconut sugar were hygroscopic even though the dried amorphous coconut sugar was more hygroscopic than the conventional coconut sugar powder [2].

Common problems that occur in food powders during storage that contribute to quality and functionality are caking due to water absorption during storage. Therefore, the addition of an anticaking agent is needed to maintain the powder stability [3]. The mechanisms of the anticaking agent's function were explained by (1) comparing it with the host powder for moisture, (2) creating moisture-protective barriers on the surface of hygroscopic particles or physical barriers between particles, (3) smoothing surfaces to eliminate interparticle friction, and (4) inhibiting crystal growth important in solid bridge formation [4]. Some of the anticaking agents used in food powder were tricalcium phosphate (TCP), silicon dioxide, calcium stearate, etc. [5]. TCP is commonly used in sugar, salt, and spices [5]. The concentration of the anticaking agent used was in the range of 1-2% [5]. Lipasek et al. [4] reported the use of an anticaking agent on deliquescent material (sucrose, sodium chloride, fructose, and citric acid) and the result that the addition of the anticaking agent resulted in reducing the moisture uptake and delaying the deliquescent point. Moreover, Nurhadi and Roos [6] added an anticaking agent to amorphous dried honey powder which resulted in reduced hygroscopicity and increased flow ability of the powder. The research to study the effect of the anticaking agent on the same material but with different structures has not been done yet. Thus, the aim of the recent work was to compare the properties of coconut sugar produced with two different methods, conventional and vacuum drying, having predominantly crystalline and amorphous structure, respectively, with the addition of the anticaking agent tricalcium phosphate.

## 2. Material and Methods

### 2.1. Materials

Coconut sap was obtained from Kertamukti village, Pangandaran District, West Java, Indonesia (170 km from the lab). Previously, before being delivered to the laboratory, the coconut sap had been boiled and then stored in a closed container. During transportation, the sample was kept cool in an ice box. In the lab, the coconut sap was kept frozen (GEA Chest Freezer, China) at -28°C and later thawed at room temperature before being used in further treatments. Maltodextrin DE 10-12 (Qinhuangdao Lihua Starch Co., Ltd., China) was used as drying aid. Tricalcium phosphate as the anticaking agent was obtained from PT Tigaka Distrindo Perkasa (Jakarta, Indonesia). The chemicals for water sorption determination in this study were lithium chloride (LiCl), magnesium chloride (MgCl_2_), potassium carbonate (K_2_CO_3_), magnesium nitrate (Mg(NO_3_)_2_), sodium nitrite (NaNO_2_), sodium chloride (NaCl), potassium iodide (KI), and potassium sulphate (K_2_SO_4_) (Merck, Germany). Silica gel and aluminium foil packaging were also used to complete the experiment.

#### 2.1.1. Coconut Sugar Production by the Conventional Method

Coconut sap was heated in a pan and continuously stirred using a spatula until it was boiling (110°C). After the coconut sap was boiled, the stirring process was speeded up to achieve a high viscosity until granules were formed [2]. The sample was then ground to reduce its size using a grinder (Getra IC-044, Indonesia) and then sieved with a 60-mesh sieve to get a homogeneous size of coconut sugar. The anticaking agent (TCP) was then added to the coconut sugar powder at different concentrations, viz., 0%, 0.5%, and 1% (per weight).

#### 2.1.2. Coconut Sugar Production by the Vacuum Drying Method

The coconut sugar powder was dried using a vacuum dryer (Binder VD 23, Tuttington, Germany), and the drying condition followed the method developed by Nurhadi et al. [2]. First, the coconut sap was mixed with maltodextrin (50% per total solid) using a magnetic stirrer on a hot plate (Thermo Scientific Cimarec™ Stirring Hotplate, USA). Previously, the solid content of the coconut sap solid was determined by a refractometer (Atago, Japan). Water was then added to the solution to reach a total solid concentration of 40%. The solution was then poured into a silicone baking tray with a thickness of ±3 mm. The temperature for vacuum drying was set to 70°C for 6 hours with absolute pressure of 5 mmHg [7]. After the coconut sap was dried, the samples were put into a desiccator to reduce the temperature to ambient temperature. The dried sample was ground to reduce its size using a grinder and then sieved using a 60-mesh sieve to get homogeneous size of coconut sugar. Then, the anticaking agent TCP was added into the resulting coconut powder at the same concentrations as in the previous experiment.

The amorphous content of both types of coconut sugar were measured by X-ray diffraction (XRD D8 Advance Bruker, Germany) and resulted 75.6% and higher than 90% for amorphous than crystalline coconut sugar, respectively. These findings complied with those in a previous research as reported by Nurhadi et al. [2].

### 2.2. Methods

#### 2.2.1. Water Sorption Isotherm (WSI)

Water sorption isotherm was determined using the static gravimetric method. Seven saturated salts were prepared to vary the relative humidity of the desiccator. The salts used were LiCl, MgCl_2_, K_2_CO_3_, NaNO_2_, NaCl, KCl, and K_2_SO_4_ (Merck, Germany), resulting in water activities of 0.14, 0.23, 0.45, 0.65, 0.75, 0.84, and 0.95, respectively. The analysis was carried out in triplicate. Samples of 1 g coconut sugar powder were weighed into vials and equilibrated over a saturated solution. The samples were weighed periodically during three weeks with a constant temperature (fluctuation 25 ± 1°C) [8]. Then, the water content of samples was measured by drying the samples in an oven at 100°C for 6 h. The Guggenheim-Anderson-de Boer (GAB) equation was used to relate water activity (*a*_w_) and moisture content.

(1) $$\begin{array}{c} X=\frac{X_{\text{m}}\cdot C\cdot Ka_{\text{w}}}{\left(1-Ka_{\text{w}}\right)\left(1+\left(C-1\right)Ka_{\text{w}}\right)}, \end{array}$$

where *X* is the water content (g water/g dry solid), *a*_w_ is the water activity, *X*_m_ is the monolayer water content, and *C* and *K* are constants.

#### 2.2.2. Particle Size Analysis (PSA)

The particle size of the sample powder was measured using a particle size analyser (Beckman Coulter, LS, USA). The particle size was expressed as the mean volumetric size [9]. The samples were placed in a test tube and dispersed using a solvent. TCP was dispersed with aquadest, and coconut sugar powder was dispersed with isopropyl alcohol [10]. The tube was then inserted into the PSA device, and the control setting was done with a computer software. The PSA data obtained was a particle distribution graph. After obtaining the particle size, the hypothetical area of coconut sugar powder and anticaking agent was calculated using the equation supplied by Earle [11] as follows:

(2) $$\begin{array}{c} A=\frac{6\;\lambda \;w}{\rho \;D}, \end{array}$$

where *A* is the area of particles, *λ* is the shape factor (1.75), *w* is the particle mass, *ρ* is the particle density, and *D* is the diameter of particles from PSA result.

The particle density of coconut sugar and anticaking agent was determined with a pycnometer (Pyrex Iwaki 2 ml). Coconut powder of known weight was filled into the pycnometer up to 2/3 of its volume. Isopropyl alcohol was then added to fill up the test volume of pycnometer until there were no more air bubbles. The pycnometer was left for 30 minutes at 25°C. Then, the pycnometer was weighed. The particle density was calculated as follows:

(3) $$\begin{array}{c} \rho_{s}=\frac{\left(m_{s}-m_{o}\right)\rho }{\left(m_{1}-m_{o}\right)-\left(m_{s1}-m_{s}\right)}, \end{array}$$

where *m*_*s*_ is the weight of the pycnometer filled with the powder, *m*_*o*_ is the weight of the empty pycnometer, *ρ* is the density of the liquid (isopropyl alcohol), *m*_1_ is the weight of the pycnometer filled with the liquid, and *m*_*s*1_ is the weight of the pycnometer filled with both the solid and the liquid [12].

#### 2.2.3. Scanning Electron Microscopy (SEM)

The surface morphology of coconut sugar powder microspheres was examined by means of JSM-IT300 InTouchScope™ Scanning Electron Microscope from Japan using a tilt angle of 40° and an accelerating voltage of 10 kV (modification from Hollenbach et al. [13]).

#### 2.2.4. Hygroscopicity Rate

Hygroscopicity was expressed as rate of water absorption by sample during storage at high relative humidity condition. Coconut powder (approximately 0.5 g) was placed in plastic vials and equilibrated over a saturated solution of NaCl with relative humidity (RH) of 75%. The weight change of sample was recorded at certain intervals for 4 hours [14].

#### 2.2.5. Angle of Repose

**Figure 1:**
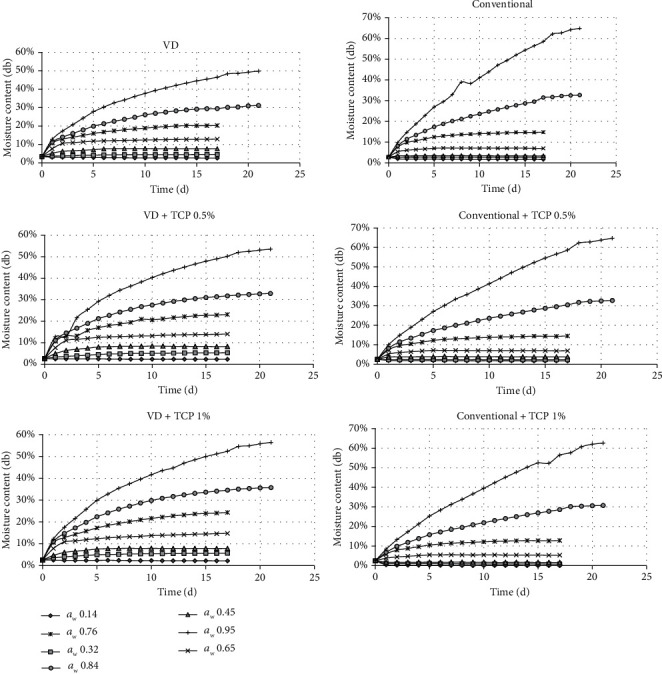
The coconut sugar powder moisture content exposed to different relative humidities during WSI experiment (db = dry basis).

**Figure 2:**
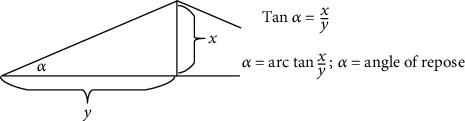
Static angle of repose.

The angle of repose (Figure 1) is a parameter commonly used for the determination of flow ability of powder. The simplest method is “poured” angle method. Firstly, 10 grams of the sample was put in a “Buchner funnel” with the open-end conditions closed. Next, the bottom of the funnel was opened and the sample was allowed to fall to a flat surface to form a balanced stack [5]. The pouring of the sample is stopped when the heap reaches a predetermined height or width. Then, the angle of repose (*α*) was calculated as follows in Figure 2; the angle of repose is measured by the inverse tangent (arctan) rule at which the average radius of the formed conical shape and the maximum height of the heaped material are measured, and then, the angle of repose is determined as the arctan of the maximum height to average radius ratio.

#### 2.2.6. Colour Analysis

Colour characteristics of coconut sugar powder were determined with a spectrophotometer (Konica Minolta CM-5 Sensing Singapore Pte Ltd). In the standard method, the spectrophotometer was used and the results were expressed as *L*∗, *a*∗, and *b*∗ (*L* is the lightness; black, *L* = 0; white, *L* = 100; +*a* is redness, −*a* is greenness; +*b* is yellowness, −*b* is blueness).

## 3. Results and Discussion

### 3.1. Water Sorption Isotherm

The change of water content during storage for both coconut sugar powders produced by the conventional and vacuum drying methods with/without the addition of TCP is presented in Figure 1. The curve demonstrated the increased water content until reaching its equilibrium water content at various water activities. The coconut sugar powder obtained by the conventional method showed lesser water sorption at each *a*_w_ compared to the coconut sugar powder from vacuum treatments.

**Figure 3:**
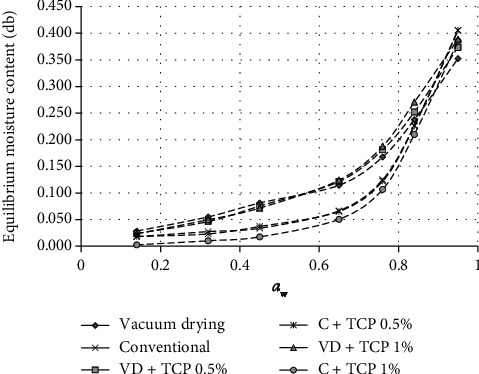
Water sorption isotherm of conventional and vacuum-dried coconut sugar (VD = vacuum drying, C=conventional).

Increasing TCP concentration impacted the time to reach the equilibrium state in the WSI experiment. Both vacuum-dried and conventional coconut sugar powder showed the same trend at increased TCP concentrations (Figures 1 and 3). This might be because increasing TCP might improve the ionic dipole in Ca^2+^ which causes a decrease in water adsorption capacity. Increasing the number of TCPs will increase the number of dipole ion bonds, and as a result, the addition of TCP 1% will reduce the water adsorption capacity more as compared to the addition of TCP 0.5%. Then, the equilibrium water content data for its corresponding *a*_w_ were fitted into the mathematical model of the Guggenheim-Anderson-de Boer (GAB) model. The GAB model is considered the best fit for food materials with a wide range of water activity and was used to correlate the WSI data. The results of the fitting procedure of the GAB model to the experimental data of equilibrium moisture content at different water activities are presented in Table 1. The value of the monolayer moisture content (*X*_m_) is of particular interest since it indicates the amount of water that is strongly adsorbed at specific sites on the food surface, and it is considered as the optimum value to assure food stability, especially microbial stability [15]. The *X*_m_ value of coconut sugar powdered by the vacuum drying method was higher than that by the conventional method, and the *X*_m_ values decreased as TCP concentration was added for both types of coconut sugar powder.

From Figure 3, it could be seen that the vacuum-dried coconut sugar with a predominant amorphous structure showed higher water sorption than the conventional one. Amorphous sugar adsorbs more water than its crystalline structure [16]. The WSI of crystalline structure showed a “J shape” with a deliquescent point which corresponds to the phase transition from solid to saturated liquid [16]. From Figure 3, the addition of the anticaking agent seemed to decrease water sorption for both types of coconut sugar. The anticaking agent might cover the hygroscopic surface of its host powder, resulting in less water absorption from the environment by the host [5].

**Figure 4:**
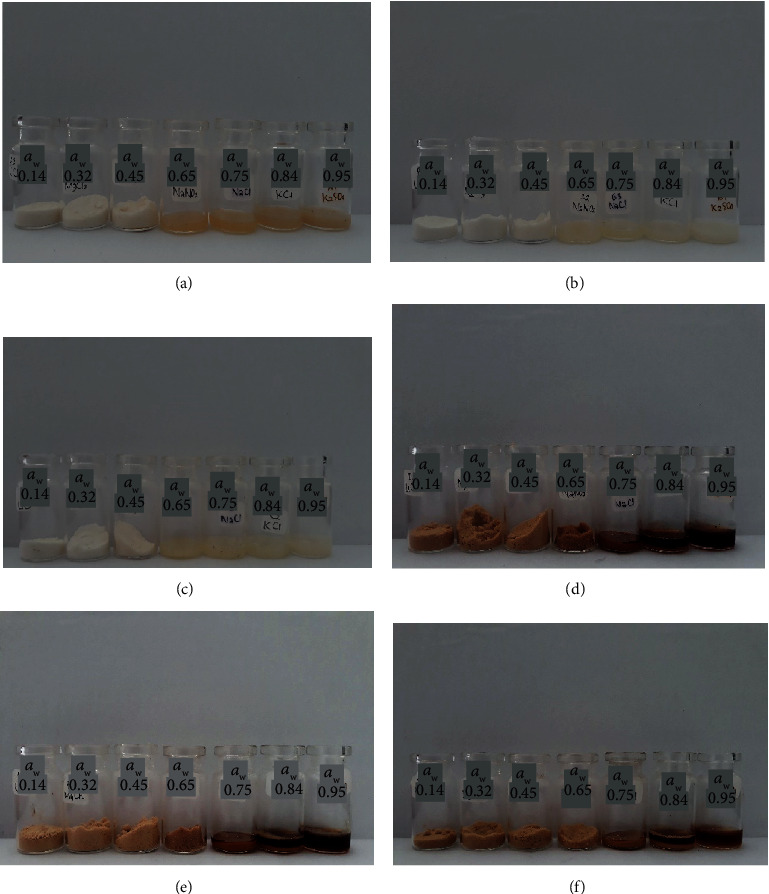
The coconut sugar stored at various water activities. (a–c) Vacuum-dried sugar without the addition of TCP, with TCP 0.5%, and with TCP 1%, respectively. (d–f) Conventional sugar without the addition of TCP and with the addition of TCP 0.5% and TCP 1%, respectively.

The addition of the anticaking agent might also inhibit caking phenomena in the amorphous sugar [2]. Caking was basically the recrystallization of amorphous sugar structure, and it was initiated by water sorption [17]. From Figure 4, the normal vacuum-dried coconut sugar started to cake at *a*_w_ 0.65 which was indicated by the forming of hard texture of sugar, while this did not occur in vacuum-dried coconut sugar with TCP addition even at a higher relative humidity. The covering of the host powder particle with TCP seemed to inhibit the form of sinter bridges between host particle powders, thus preventing the caking [5].

While caking was not observed in the crystalline coconut sugar formed by the conventional method, the deliquescence started to occur at *a*_w_ 0.75 for both normal crystalline sugar and the sugar with TCP addition. Lipasek et al. [4] reported that the anticaking agent might delay the deliquescence point.

### 3.2. Particle Size Analysis (PSA)

**Figure 5:**
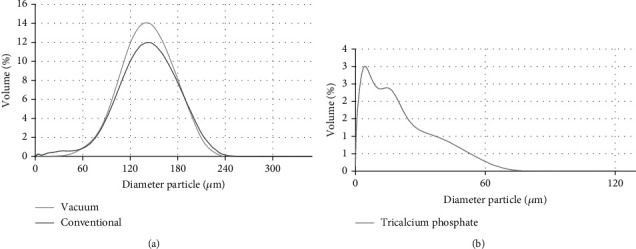
Distribution particle size of (a) conventional and vacuum-dried coconut sugar powder and (b) tricalcium phosphate.

One of the mechanisms of the anticaking agent in maintaining the storage stability of hygroscopic host powder is by covering its hygroscopic surface area thus protecting the host from absorbing water [5]. Because the amount of the anticaking agent allowed is very small (less than 2% per total weight), to be able to cover the surface area of the host material, the anticaking agent should have a very low particle size. The lower the particle size, the higher the resulting surface area. From Figure 5, it could be seen that the anticaking agent TCP had a lower molecular weight compared to both coconut sugars as host. The particle size and surface area of TCP and the two types of coconut sugar can be seen in Table 2.

We proposed that the addition of anticaking TCP would cover the surface area of coconut sugar as a host material to inhibit the absorption of moisture from the environment. The surface area calculation was determined as explained by Earle [11]. The shape factor chosen was 1.75 for ground material compared to 1 for cube and sphere [11]. The calculation of surface area for both types of the sugar and TCP are presented in Table 2. From Table 2, it could be seen that the addition of the anticaking agent up to 1% could not cover completely the surface area of the coconut as the host powder.

The increasing concentration of TCP increased the resulting surface area. TCP should be added hypothetically to cover completely at a concentration of 1.59% and 1.53% for the vacuum-dried coconut sugar and conventional coconut sugar, respectively. From Table 2, the TCP density is 0.317 g/ml, which is lower than the density of coconut sugar. Therefore, TCP would be at the top of the host surface and compete with the host powder to adsorb moisture from the environment [18].

### 3.3. Scanning Electron Microscopy (SEM)

**Figure 6:**
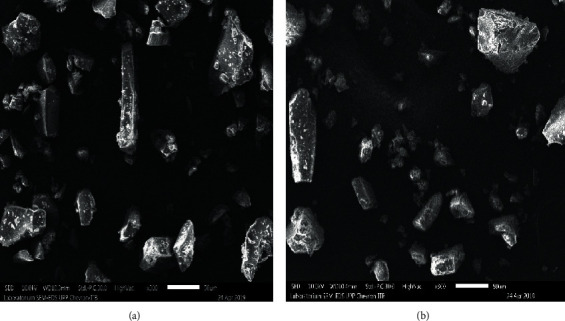
Scanning electron micrographs of vacuum-dried coconut sugar powder particles (a) without and (b) with the addition of 1% tricalcium phosphate.

**Figure 7:**
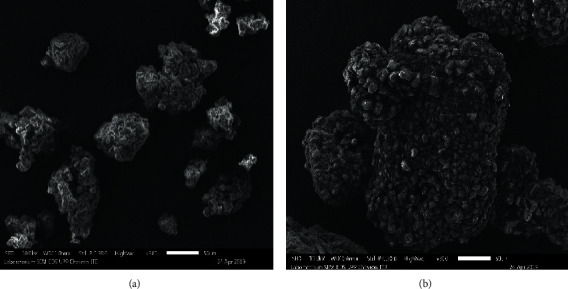
Scanning electron micrographs of conventional coconut sugar powder particles (a) without and (b) with the addition of 1% tricalcium phosphate.

Photomicrographs of coconut sugar powder with two methods and tricalcium phosphate (TCP) are shown in Figures 6 and 7. TCP has a very fine size and tends to aggregate and form soft agglomerates with a very nonuniform size [13]. As previously stated, one of anticaking agent mechanisms is by covering the surface area of the host material which in this case is the coconut sugar [5]. The presence of TCP on the surface of coconut sugar powder is clearly seen on the sample from the conventional method compared to that from the vacuum drying method (Figures 6 and 7). From Figure 7, the TCP with the finer size stuck on the surface of conventional coconut sugar. The covering of the host powder with anticaking TCP would prevent the host from absorbing moisture from the surroundings and consequently delay the powder caking.

### 3.4. Hygroscopic Rate

**Figure 8:**
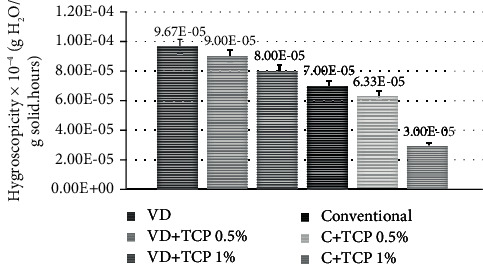
Hygroscopicity rate of coconut sugar powder produced by traditional and vacuum drying methods.

From Figure 8, the hygroscopicity of vacuum-dried coconut sugar powder was higher than that of conventional method coconut sugar powder. This is due to the predominantly amorphous structure of the coconut sugar obtained from vacuum drying [2]. The amorphous material has a greater pore size than the crystalline material thus having greater water sorption [19]. As can be seen from the WSI curve (Figure 3), amorphous coconut sugar adsorbs water in higher amounts than the conventional one. The amorphous structure has molecules that are not arranged regularly, are more open, and have a large volume. Therefore, it is easier to bind to water from the environment. Meanwhile, properties of crystalline structure are nonhygroscopic, stable, and free flowing. Hence, its water absorption only occurs on the external surface of the crystal [20].

The addition of an anticaking agent could decrease the hygroscopicity of both types of coconut sugar. The higher the TCP added, the lower the hygroscopicity. Coconut sugar powder with a higher addition of TCP concentration would produce more covered area to the host (coconut sugar) which made it difficult for water vapor to be adsorbed into the material. From Figure 8, it seems that the addition of the anticaking agent gave more significant effect on decreasing the hygroscopicity of the conventional coconut sugar with predominant crystalline structure than the vacuum-dried amorphous one.

### 3.5. Angle of Repose

Free flowing and granular materials when poured through a funnel on a flat surface produce a cone with a small angle of repose of 35° or less, while cohesive powders, in contrast, have a higher angle of repose (higher than 55°) [5]. From Table 3, all the coconut sugar powders showed free flowing properties with angle of repose value in the range of 26.1–32.8°. The result seemed to give the same trend as the result of hygroscopicity, where the more hygroscopic the sample, the less the ability of the sample to flow. The addition of anticaking agents such as TCP could reduce the angle of repose from the structure of amorphous and crystalline sugar powder (Table 3). The smaller the angle of repose, the higher the flowability of the product. The addition of the anticaking agent on honey powder also reportedly increased its flow ability [2]. The mechanism of the anticaking agent in increasing the flow ability might be due to its capacity to decrease internal friction within the host material for both the amorphous and crystalline material, and as a result, there were less restriction for the materials to flow [4, 5, 16].

### 3.6. Colour Analysis

**Figure 9:**
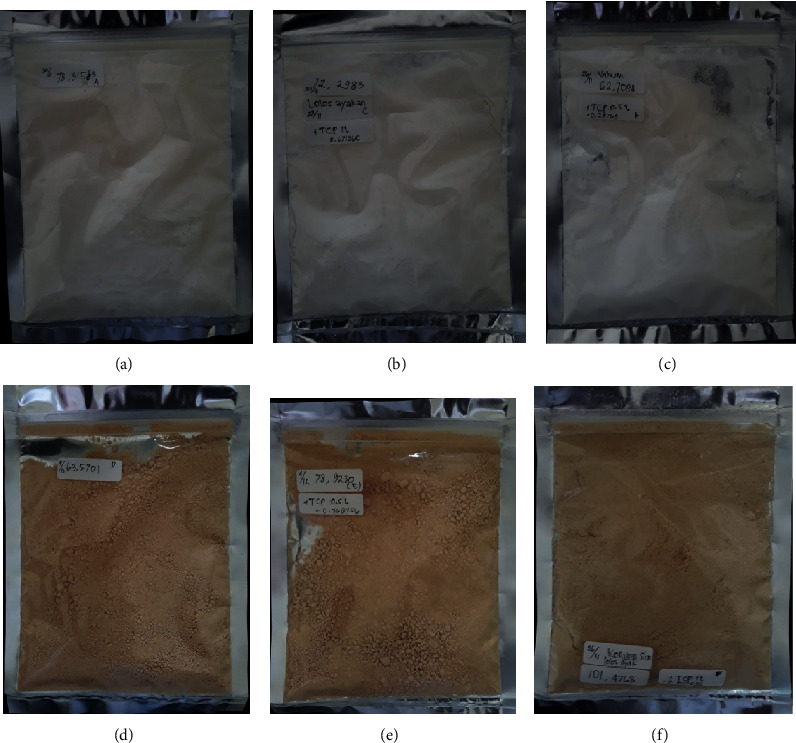
Coconut sugar powder. (a–c) Vacuum-dried sugar without the addition of TCP, with TCP 0.5%, and with TCP 1%, respectively. (d–f) Conventional sugar without the addition of TCP and with the addition of TCP 0.5% and TCP 1%, respectively.

The colour of coconut sugar for all treatments can be seen in Figure 9. The coconut sugar obtained from vacuum drying showed brighter colour due to the addition of maltodextrin (50% per total weight). The colour of maltodextrin itself is white. The darker colour of coconut sugar powder that was produced by the conventional method might be due to Maillard reaction and caramelization occurring during processing at high temperature (Table 4). The Maillard reaction occurs due to the reaction between reducing sugar and amino acids, while the caramelization reaction occurs due to the interaction of sugars at high temperatures (80°C) [21].

The addition of the anticaking agent seemed not to affect the colour of the coconut sugar. The anticaking agent TCP has white colour, but the concentration used was very small (maximum 1% per total weight) thus not making a significant colour difference.

## 4. Conclusions

Coconut sugar was obtained by two different methods, conventional and vacuum drying. The conventional coconut sugar had a predominantly crystalline structure, while the vacuum-dried coconut sugar had mainly an amorphous structure (75.6%). The anticaking agent (tricalcium phosphate (TCP)) was added to maintain the stability of coconut sugar during storage and to increase its flow ability. The TCP addition seemed to affect the water sorption of both types of coconut sugar by decreasing their monolayer water content (*X*_m_). The addition of the anticaking agent (TCP) resulted in decreased hygroscopicity and increased flow ability for both types of coconut sugar. The mechanism of TCP to maintain stability of coconut sugar might be related to covering the surface area of the host material (coconut sugar) by TCP thus preventing the host from water absorption. The TCP addition had a more significant effect on decreasing the hygroscopicity of the conventional coconut sugar with a predominantly crystalline structure than the amorphous vacuum-dried coconut sugar.

## Figures and Tables

**Table 1:** Estimated GAB parameters of coconut sugar powder produced by the conventional method and the drying methods.

Samples	GAB model			
	*X*_m_	*K*	*C*	*R* ^2^
Vacuum drying (VD)	0.183	0.938	2.245	0.973
VD+TCP 0.5%	0.158	1.000	2.269	0.970
VD+TCP 1%	0.099	0.894	2.209	0.976
Conventional	0.042	0.910	2.166	0.976
Conventional+TCP 0.5%	0.035	0.909	2.148	0.977
Conventional+TCP 1%	0.032	0.926	2.110	0.977

**Table 2:** The hypothetical area of coconut sugar powder produced by vacuum drying and conventional method and tricalcium phosphate (TCP).

Sample	Weight (g)	*D*	*ρ* (g/ml)	*A* (m^2^)	% area cover	
					Vacuum	Conventional
Vacuum	100	137.7 *μ*m	1.418	5.379	—	—
Conventional	100	126.7 *μ*m	1.5997	5.181	—	—
TCP 0.5%	0.5	9.809 *μ*m	0.317	1.688	31.4	32.6
TCP 1%	1	9.809 *μ*m	0.317	3.376	62.8	65.2

**Table 3:** Angle of repose with the addition of TCP.

Samples	Angle of repose (°)
Vacuum drying	32.8 ± 0.007
VD+TCP 0.5%	30.9 ± 0.022
VD+TCP 1%	31.0 ± 0.055
Conventional	27.3 ± 0.027
Conventional+TCP 0.5%	26.1 ± 0.02
Conventional+TCP 1%	25.0 ± 0.007

**Table 4:** Colour analysis with the addition of TCP.

Samples	*L*∗	*a*∗	*b*∗
VD	95.26 ± 1.26	0.71 ± 0.005	9.63 ± 1.02
VD+TCP 0.5%	96.32 ± 0.32	0.70 ± 0.078	7.41 ± 0.85
VD+TCP 1%	96.22 ± 0.29	0.69 ± 0.16	7.31 ± 0.41
Conventional	84.20 ± 1.12	4.85 ± 0.90	19.00 ± 1.85
Conventional+TCP 0.5%	84.53 ± 1.10	4.14 ± 0.54	18.89 ± 0.82
Conventional+TCP 1%	83.69 ± 0.69	3.47 ± 0.69	18.86 ± 0.70

## Data Availability

The data used to support the findings of this study are available from the corresponding author upon request.
